# Trafficking of Neuronal Two Pore Domain Potassium Channels

**DOI:** 10.2174/157015910792246146

**Published:** 2010-09

**Authors:** Alistair Mathie, Kathryn A Rees, Mickael F El Hachmane, Emma L Veale

**Affiliations:** Medway School of Pharmacy, Universities of Kent and Greenwich at Medway, Central Avenue, Chatham Maritime, Kent ME4 4TB, UK

**Keywords:** Two pore domain potassium channel, TASK, TREK, p11, 14-3-3, endoplasmic reticulum, trafficking, neuronal membrane, K2P.

## Abstract

The activity of two pore domain potassium (K2P) channels regulates neuronal excitability and cell firing. Post-translational regulation of K2P channel trafficking to the membrane controls the number of functional channels at the neuronal membrane affecting the functional properties of neurons. In this review, we describe the general features of K channel trafficking from the endoplasmic reticulum (ER) to the plasma membrane *via *the Golgi apparatus then focus on established regulatory mechanisms for K2P channel trafficking. We describe the regulation of trafficking of TASK channels from the ER or their retention within the ER and consider the competing hypotheses for the roles of the chaperone proteins 14-3-3, COP1 and p11 in these processes and where these proteins bind to TASK channels. We also describe the localisation of TREK channels to particular regions of the neuronal membrane and the involvement of the TREK channel binding partners AKAP150 and Mtap2 in this localisation. We describe the roles of other K2P channel binding partners including Arf6, EFA6 and SUMO for TWIK1 channels and Vpu for TASK1 channels. Finally, we consider the potential importance of K2P channel trafficking in a number of disease states such as neuropathic pain and cancer and the protection of neurons from ischemic damage. We suggest that a better understanding of the mechanisms and regulations that underpin the trafficking of K2P channels to the plasma membrane and to localised regions therein may considerably enhance the probability of future therapeutic advances in these areas.

## 1. INTRODUCTION

Two pore domain potassium (K2P) channels encode background, or leak, K currents which are essential players in the regulation of the resting membrane potential and excitability of many mammalian neurons. The 15 members of the K2P channel family can be divided into 6 subfamilies on the basis of their structural and functional properties, namely the TREK, TASK, TWIK, THIK, TRESK and TALK subfamilies [1, 27, 33, 44]. The subfamilies vary in their amino acid sequence as well as in tissue distribution and pharmacology, but two characteristic features of all K2P channels are that they are not voltage-gated and they are not inhibited by the classical potassium channel blocking agents, TEA and 4-AP [44].

The activity of K2P channels is regulated by a diverse array of pharmacological and physiological mediators [13, 44, 49, 68] and by a large number of neurotransmitter activated pathways [48]. Evidence is accumulating for the potential importance of targeting and altering the activity of K2P channels in a number of therapeutic situations in the nervous system, including neuroprotection, neuropathic pain, depression, anesthesia and epilepsy [4, 5, 29, 43, 68]. Since the activity of K2P channels is of such importance in determining neuronal excitability and cell firing [8, 50], it follows that any post-translational regulation of trafficking which significantly alters the number of channels and therefore current density at the neuronal membrane would have profound effects on the functional properties of these neurons.

In this review, we will consider current evidence concerning the trafficking of K2P channels to the neuronal membrane and their localisation therein. Whilst there are some general mechanisms that apply to many ion channels, for the most part, evidence suggests that each channel type has different processes which dominate these events. There are two particular processes regarding K2P channel trafficking for which most evidence exists. These are the regulation of trafficking of TASK channels from the endoplasmic reticulum (ER) or their retention within the ER [26, 56, 57, 64, 65, 95, 96] and the localisation of TREK channels to particular regions of the neuronal membrane [72, 73]. We begin with a brief, general summary of K channel trafficking; particularly K_V_ channel trafficking for which most evidence exists; to set out some important considerations, then focus on the K2P channels themselves.

## 2. POTASSIUM CHANNEL TRAFFICKING: GENERAL FEATURES

### 2.1 First Step: from the Nucleus to the ER 

Whilst functional ion channels are generally considered as originating in the ER, the formation process begins earlier. mRNA for the channel protein is made and exported from the nucleus to the cytosol. In the cytosol, the mRNA associates in a complex with cytosolic ribosomes and tRNA and undergoes translation. As the peptide is translated from the peptidyl transferase centre and elongates, it travels along a long (100 Å) tunnel within the ribosome, coined the “birth canal” (for a detailed review see [18]). The ribosomal tunnel itself is thought sufficiently large enough in width (10-20 Å) to permit secondary structure, but not tertiary. Once translation is complete, the entire peptide/ribosome complex is targeted to the ER membrane where synthesis can continue. This targeting of the peptide/ribosome complex is thought to involve a signal sequence within a hydrophobic helical region of the n-terminus of the channel. This signal motif is thought to bind with a signal recognition particle (SRP), which, in turn, binds to a receptor on the cytoplasmic side of the ER membrane. This process takes the order of minutes. Some very nice work with the K_V_ channel, K_V_1.3, has shown that these subunits use several transmembrane domains for targeting of the complex to the ER, with the second transmembrane segment (S2) acting as the SRP signal motif [87]. Thus the topology of the channel even at this early stage in development seems to be important in determining its traffic to the ER, at least in the case of K_V_1.3, and highlights a high degree of regulation of protein translation at a very early stage in production. 

### 2.2 Assembly in the ER

Once the peptide/ribosome complex reaches the ER, it binds to a multi-protein complex known as the translocon, which forms an aqueous pore, permitting passage across the lipid bilayer of the ER membrane. The peptide eventually emerges from the ribosome into the lumen of the ER [32]. It is in the translocon that the protein topology - the parts of the protein that will be cytosolic, transmembranous or extracellular is determined (see [17]). Most ion channels do not exist as individual subunits, but as dimers or tetramers. So at some point these subunits must associate to form a functional ion channel. There is much speculation as to where this association occurs, but the general consensus is that the ER is the most likely site for this. K_V_ channels, which form tetramers, have been shown to couple two dimers together, rather than sequentially combine subunits [86]. Oligomerization of K_V_ channels occurs *via *the highly conserved tetramerisation domain (T1) in the N-terminus [36, 42]. However, mutated channels missing the T1 domain, can still assemble *via *a transmembrane associated domain, but the rate, efficiency and accuracy of this is much lower [18]. Not all recognition domains for oligomerisation of K channels are found in the N-terminus of channels, but, instead some reside in their C-terminus [75]. Some channels will not assemble properly in the ER, so misfolded and misassembled channels are, generally, retained here. Residues will be exposed that shouldn’t be exposed and these might encourage aggregation of misfolded proteins for ER retention and degradation.

### 2.3 Onwards from the ER 

As the peptide emerges from the translocon it is extruded into the ER lumen, where amino acids exposed to the cytosol are able to associate with cytosolic proteins, such as chaperones and auxiliary subunits, which assist in the folding, modification and trafficking of the ion channel protein. In this context, chaperone proteins can be defined as proteins which form a transient association with ion channels, and which help to prevent mis-folding of the newly formed channel proteins, act to retain incorrectly folded proteins for degradation or act to promote forward trafficking towards the membrane. Auxiliary subunits, by contrast, associate more permanently with the ion channels to regulate both the function and the trafficking of the channel. However, the distinction between the two is often blurred and one often sees the term “auxiliary protein” to describe proteins which, strictly speaking, act as chaperones rather than auxiliary subunits. 

### 2.4 Auxiliary Subunits

For K channels, a number of different auxiliary subunits have been identified. These auxiliary subunits can either associate with the N or C-terminus of the channel or intercalate between the pore forming subunits. The most documented K channel auxiliary subunits are the β-subunits which associate with certain K_V_ channels to assemble, modulate and traffic the channels [10, 24, 28, 77]. Different isoforms of these β-subunits exist, which associate with different K_V_ channels in the ER [54]. The major β-subunit isoforms are K_V_β1 & K_V_β2. 

Another form of β-subunit is the K_V_-Channel Interacting Protein (KChIP) which has been shown to associate with the n-terminus of K_V_4 channels [3, 34, 47, 74, 78, 94]. The binding of KChiP to hydrophobic residues in the N-terminus (7-11) and hydrophilic residues (71-90) promotes surface trafficking of K_V_4.2 by masking an ER retention signal [74]. In the absence of KChIP, K_V_4 channels were found to accumulate in the ER.

KChAP (or K Channel Associated Protein) has been suggested to have a chaperone role (although sometimes it is classified as an auxiliary subunit). KChAP binds to the N-terminus of the α subunit of K_V_1 & K_V_2 family members and increases cell surface expression, without modifying the biophysical properties of the channels [37, 90]. KChAP has also been shown to stabilise the K_V_α-K_V_β complex, by binding to the C-terminus of K_V_β subunits. Similar to KChAP, the G protein β γ (Gβγ) has been shown to stabilise a K_V_1.1-K_V_β complex [31].

One other well known K channel auxiliary subunit is the sulfonyl urea receptor (SUR) which both modulates and traffics the inward rectifying channel K_IR_6.2, together forming functional K_ATP_ channels. The SUR associates with K_IR_6.2 in the ER and early Golgi *via *regions in the first transmembrane segment (M1) and the cytosolic N-terminus [76]. 

### 2.5 Chaperone Proteins for Membrane Trafficking

A bewildering array of chaperone proteins exist, involved in trafficking proteins around cells and to particular regions of cells. For ion channels, interest has centred on those chaperones which assist with trafficking to and from the membrane, those that target the channels to particular regions of the membrane and those involved in recycling of channels from the membrane. Rather than cover each exhaustively, we focus here on those chaperone proteins with identified roles in the trafficking of TASK K2P channels (see Table **1**).

The coatomer protein complex 1 (COP1) and 14-3-3 chaperone system is common to several membrane proteins including KA2 kainate receptors and TASK K2P channels (see section 4.1) [89]. COPI coated vesicles are formed, which are major protein carriers in the early endocytic pathway, controlling Golgi apparatus to ER retrograde transport [6]. 14-3-3 proteins are a large family of adaptor proteins with roles in many cellular processes including apoptosis, metabolism and membrane protein trafficking (see [52]). 14-3-3 proteins are particularly involved in intracellular trafficking and the promotion of forward trafficking between the ER and the plasma membrane. COP1 and 14-3-3 often act in competition to retain channels in the ER or promote their trafficking towards the plasma membrane (see later). 

Another chaperone protein that has been implicated in the trafficking of TASK channels is p11, also known as s100A10 or annexin II light chain. p11 is a member of the s100 family of E-F hand proteins and it is an adaptor protein that binds to annexin 2 and other substrates to play a role in endocytosis, membrane trafficking and actin polymerisation [66, 85]. p11 has been shown to target channels to specific microdomains in the plasma membrane and has also been linked to the translocation of Na_V_1.8, ASIC and TRPV5/6 channels and the 5HT1b receptor [26, 84].

### 2.6 Binding Motifs

Chaperone proteins must interact physically with the channels they partner; so much work has centred on identifying common binding motifs – sequences of amino acids on the channel to which chaperone proteins might bind. From such studies a number of common sequences have emerged [38, 82]. For example, specific amino acid sequences known as retention motifs dictate whether a membrane protein is detained in/returned to the ER or transported to the plasma membrane [45, 46]. Channels tend to contain several motifs that may compete with each other. A common ER retention motif is the ‘di-lysine’ motif (KKxx). This motif is common to many potassium channels and is a major regulatory mechanism to ensure that only properly assembled ion protein complexes are transported. The ‘masking’ of ER retention motifs and trafficking to the membrane occurs only when the protein is properly folded, as demonstrated for example, for the K_ATP_ channel [93]. ‘Dibasic’ motifs can also cause ER retention through interaction with the COPI complex (introduced above). 

Another ER retention signal, KDEL, targets proteins for Golgi to ER recycling, whilst other forward trafficking motifs for transport from ER to Golgi, e.g. FYCENE for K_IR_2.1, and dileucine motifs, present in many K channels [38, 82].

### 2.7 To the Golgi Apparatus then the Membrane

From the ER, channel proteins enter the Golgi apparatus en route to the plasma membrane. Glycosylation occurs here, which is an important step for surface expression of many channels such as EAG1, K_ATP_, K_V_1.4 and other K_V_1s [82]. Once close to the membrane, channels seem to be inserted by a fairly conserved process. This involves SNARE mediated fusion of exocytotic vesicles with the plasma membrane. This has been well established for K_V_1.1 and K_V_2.1, for example (see [82]). In neurons targeting is highly specific (e.g. K_V_4.2 goes to distal regions of dendrites, K_V_1 channels go to juxtaparanodal region). This involves motor proteins, actin, microtubule cytoskeleton, scaffolding proteins and accessory subunits but the fine details underlying these mechanisms are poorly understood (see, for example, [38]).

Again, chaperone proteins have a major role to play in channel localisation. For example, CASK (a MAGUK protein) is implicated in targeting of K_IR_2 channels in brain and heart. CASK is known to complex with PDZ proteins (e.g. SAP97 a protein closely related to PSD95), so perhaps it acts as a scaffolding protein that anchors K channels at their target location. SAP97 also interacts with K_V_1.5, and this complex localises to lipid rafts. Disruption of cytoskeleton leads to an increase in K_V_1.5 surface expression although it has no effect on K_V_2.1. Dileucine motifs have also been suggested to play a role in the targeting of ion channels to particular membrane regions. So, for example, dileucine motifs on the C terminus promote axonal localisation for Na_V_ channels but similar motifs on the C terminus of K_V_4.2 channels promotes dendritic localisation [38]. Deletion of a dileucine targeting domain stopped K_V_4.2 being specifically targeted to dendrites and instead was found throughout the neuron [82].

Selective localisation occurs in many different ways. In addition to CASK and PDZ proteins (such as SAP97 and PSD95), actin binding proteins (such as alpha-actinin-2) are implicated in targeting and anchoring (e.g. for K_V_1.5). Actinin may also be involved in K_V_1.5 channel endocytosis and/or maintaining pools of K_V_1.5 in vesicles just below the membrane. The protein, dynamin is also implicated in K_V_1.5 expression levels. K_V_1.5 currents are increased by dynamin inhibitory peptide suggesting that dynamin stimulates tonic turnover of K_V_1.5 levels at the membrane, perhaps through clathrin-dependent or -independent endocytosis. 

After internalisation, channels must be either recycled to the membrane or degraded. Evidence is very sparse on what happens and how it happens at this stage. It has been suggested that ubiquitination of ion channels is an important step in the processes underlying K channel internalisation and recycling [82].

## 3. K2P CHANNEL TRAFFICKING

### 3.1 The Role of 14-3-3 and COP1 in TASK Channel Trafficking from the ER

Yeast 2 hybrid studies have revealed that TASK channels (TASK1, TASK3 and even the non-functional TASK5) bind to 14-3-3 proteins both in recombinant and native form [26, 64]. Mutational studies showed that only TASK channels that interacted with 14-3-3 were present at the plasma membrane [64]. All seven isoforms of 14-3-3 (β, γ, ε, ζ, η, τ and σ) bind to TASK channels, although O’Kelly *et al.* [56] showed that 14-3-3β binds with the highest affinity. 

Yeast two hybrid studies and GST-pull down assays using WT and truncated channels have also revealed the binding of COPI (the β subunit more specifically) to TASK channels [56]. The interaction between COP1 and TASK channels leads to decreased surface expression of channels and accumulation of channels in the ER. Thus COPI and 14-3-3 act in opposite ways to either promote TASK channel forward trafficking towards the membrane (14-3-3) or retain TASK channels in the ER (COPI).

There are several hypotheses that could explain how 14-3-3 and COPI interact to regulate TASK channel trafficking [52, 80]. These include “clamping”, where binding of 14-3-3 would cause a conformational change in the TASK channel to prevent binding of COP1, usually envisaged to bind to a different site in the TASK channel sequence; “scaffolding”, where binding of 14-3-3 would trigger recruitment of additional trafficking proteins which enhance TASK channel trafficking; or “masking” where 14-3-3 would bind to a particular site on the TASK channel and exclude the binding of COP1 or, indeed, other proteins to that same site. 

**Fig. (1):**
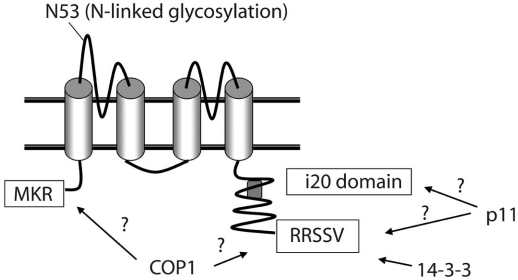
Regions of TASK1 K2P channels which interact with binding partners. Schematic representation of a TASK1 K2P channel illustrating potentially important regions of the channel for interactions with binding partners such as COP1, 14-3-3 and p11.

Of these hypotheses, the most favoured idea, until recently, for the interaction of 14-3-3 and COP1 in regulating TASK channel trafficking was clamping, so that the change in conformation induced by 14-3-3 binding was proposed to cause an inactivation of the COP1-interacting motifs [52]. Furthermore, initial experimental evidence suggested that 14-3-3 binding inhibited COP1 binding, but that the two proteins did not compete for a binding site. Rather they were suggested to bind at separate dibasic sites on TASK1 channels and that binding was ‘mutually exclusive’. COP1 was originally suggested to bind to the N-terminus of TASK channels at the dibasic motif (M)KR [56, 92] while 14-3-3 was shown to bind to TASK1 and TASK3 at the extreme C-terminus, dibasic motif (RR(K/S)SV) and, importantly, phosphorylation of the distal serine residue was required for the interaction with TASK1 [56, 79]. This led O’Kelly and Goldstein [57] to propose that, normally, COP1 is bound to the channel at the N-terminus dibasic motif (Fig. ** 1**), causing retrieval from the Golgi apparatus and subsequent retention in the ER. When 14-3-3 binds to the phosphorylated extreme C-terminus of TASK, it causes COPI to dissociate from the channel. Bound 14-3-3 inhibits the ER retention motif and forward trafficking to the plasma membrane can take place. In this way 14-3-3 is able to promote forward trafficking to the plasma membrane [57] and channel number at the cell surface is therefore increased. 

A similar mechanism has been proposed for the regulation of KA2, kainate receptor, trafficking by 14-3-3 and COP1 [89]. Furthermore, Shikano *et al.* [79] found that a motif FRGRSWTY (termed SWTY) in K_IR_2.1 channels recruited 14-3-3 isoforms, and in doing so was able to override the RKR ER-retention motif. Again, 14-3-3 binding was dependent upon phosphorylation, this time of the threonine residue in the binding motif (SWpTY).

**Fig. (2):**
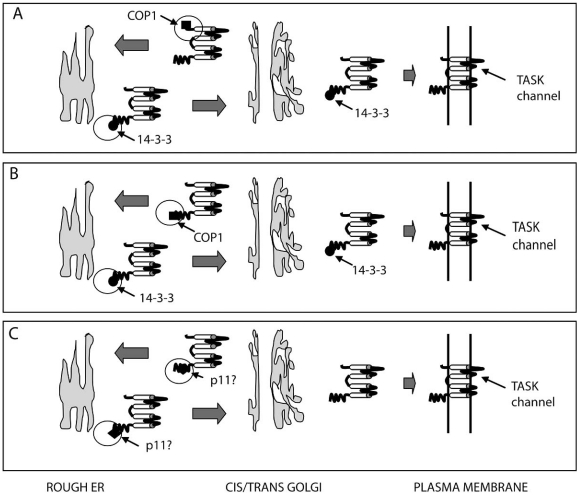
Putative trafficking mechanisms for TASK K2P channels. **A**) 14-3-3 promotes TASK channel trafficking to the membrane whilst COP1 promotes channel retention in the ER. COP1 and 14-3-3 bind mutually exclusively to different regions of the TASK channel as proposed by [57]. **B**) 14-3-3 promotes TASK channel trafficking to the membrane whilst COP1 promotes channel retention in the ER. COP1 and 14-3-3 bind mutually exclusively to the same region of the TASK channel as proposed by [95]. **C**) P11 either promotes TASK1 channel trafficking to the plasma membrane [57] or promotes retention of TASK1 channels in the ER [65] by binding to identified regions in the C terminus of the channel.

However, an impressively thorough, recent study from Zuzarte *et al.* [95] provides evidence to show that 14-3-3 binds to the extreme C terminus of both TASK1 and TASK3 to mask the retention motif and stops this region of the channel binding to COP1 (Fig. ** 1**), thereby favouring the masking hypothesis rather than the clamping hypothesis above. This study suggested that the N terminal retention signal operated independently of 14-3-3 binding, the latter being a prerequisite for trafficking of the channel to the membrane suggesting that the extreme C terminus retention signal is dominant. This is, of course, in direct contrast to the conclusions drawn by O’Kelly *et al.* [56] and O’Kelly and Goldstein [57] described above. Indeed, Zuzarte *et al.* [95] suggest that the C terminus alone (of both TASK1 and TASK3) is sufficient to bind COP1 and that the N terminus is not involved in COPI binding (see Fig. **2A**, **B**).

It has been suggested that for forward trafficking of the GABA_B_ receptor, the COPI and 14-3-3 trafficking mechanism is due to competitive binding, not a change in structure, where COP1 binding is lost when the concentration of 14-3-3 is high and vice versa [9]. 

14-3-3 has also recently been found to co localise with TRESK channels (Table **1**), although, for this K2P channel, 14-3-3 is thought to have a direct regulatory role rather than a trafficking one [14]. No other K2P channels have so far been found to colocalise with 14-3-3 or COP1, perhaps suggesting that there is not a general mechanism for K2P trafficking mediated by the interaction of these proteins.

### 3.2 The Putative Role of p11 (s100A10) in TASK Channel Trafficking

The adaptor protein, p11, has also been found to interact with TASK channels using yeast-2 hybrid assays and this has been confirmed with co-localisation studies using GST- pull down and immunoprecipitation [26, 65]. The association with TASK1 has been linked to surface expression of channels. There is, however, some debate regarding whether p11 inhibits or promotes forward trafficking. All studies to date have shown that p11 only binds to TASK1 (not to TASK3 or TASK5), and that this binding is dependent on the presence of 14-3-3. p11 cannot bind to TASK1 in the absence of 14-3-3, whilst p11 and 14-3-3 do not interact without TASK1 [26, 65]. 

Girard *et al.* [26] and O’Kelly and Goldstein [57] demonstrated that p11 promotes forward trafficking and binds at the same extreme C-terminal dibasic sequence as 14-3-3, the critical binding sequence (ascertained using mutational studies) being the last 3 amino acids; SSV (part of the 14-3-3 binding motif, above, Fig. **1**). This sequence is also a putative PDZ type 1 binding domain, however to date, no known PDZ domain proteins have been shown to colocalise with TASK1. Both groups used truncated channel studies to show that p11 interaction with TASK1 channels lead to increased channel trafficking to the plasma membrane and therefore higher functional surface expression [26, 57, but see  88].

O’Kelly and Goldstein [57] also looked at the tissue distribution of p11, and observed high levels in the brain and lung. Significantly, they found low expression in the heart, where TASK1 channels are highly expressed. In contrast 14-3-3 proteins have relatively high expression levels in all tissue types. The limited tissue distribution and dependency of p11 on 14-3-3 co-localisation led O’Kelly and Goldstein [57] to hypothesise that p11 has a partial, modulatory role in TASK1 trafficking only. Hypothetically, p11, 14-3-3 and TASK1 interact to form a ‘ternary complex’ to promote forward trafficking in a tissue-specific manner.

However, and in complete contrast, Renigunta *et al.* [65] showed that p11 inhibited forward trafficking and deletion of p11 using siRNA lead to an increase in channel density at the cell surface. This group showed that p11 binds at a separate site 80-120 amino acids from the C terminus (approximated using deletion of sequence sections and p11 binding studies), (Fig. ** 1**). The group also concluded that p11 has a ‘di-lysine’ motif within its structure that would cause the channels to be retained in the ER (similar to classical COP1 binding motifs). Furthermore, Zuzarte *et al.* [95] suggest that the observed C terminal truncation experiments, which, in their hands, reduced current amplitude of both TASK1 and TASK3 channel currents to around the same degree, might be attributable to the preclusion of 14-3-3 binding, rather than p11 interactions, particularly since TASK3 channels do not interact with p11. 

Thus, at present, there is conflicting evidence concerning the role of p11 in trafficking of TASK1 channels and suggestions that it may promote [26, 57] or inhibit [65, 95] TASK1 channel trafficking to the plasma membrane (see Fig. **2C**). p11 is found to positively influence the trafficking of other ion channels and plasma membrane proteins to the neuronal membrane, including 5-HT1b receptors, ASICa channels, Na_V_1.8 channels and TRPV5/6 channels [20, 25, 58, 84]. 

The differences in trafficking mechanism between TASK1 and TASK3 channels are highlighted by the poor surface expression of TASK1 channels in recombinant cell lines and the consequential small current recorded in comparison to the robust TASK3 current in such cells (suggesting that TASK3 membrane expression is good). Whereas in native systems TASK1 currents are often larger, suggesting that forward trafficking occurs appropriately in these cells. It remains to be seen whether interaction with p11 or some currently unknown component (lacking in recombinant systems) is involved in the proper trafficking of the TASK family in native neurons. 

### 3.3 The EDE Motif for TASK3 

A further unique sequence motif has been identified in the proximal C terminus of the TASK channel, TASK3. This di-acidic sequence (EDE) has a role in trafficking TASK3 channels to the membrane since mutation of the two glutamate residues reduces surface expression [96]. Whilst this region is suggested to be required for efficient surface expression of TASK3 channels through interactions with a functional COPII complex, it cannot overcome the strong retention signal, described above, at the extreme C terminus of the channel which is masked by 14-3-3 binding [95, 96]. A similar EDE sequence is found in TASK1 channels but its functional importance has not yet been determined.

### 3.4 Other K2P Channel Binding Partners

Relatively little is currently known concerning the mechanisms that regulate the insertion of functional K2P channels into the plasma membrane. It has however been suggested that the non-functionally expressed channels (KCNK7, TASK5 and THIK2) are so, due to stringent internal retention mechanisms [22, 71]. 

#### 3.4.1 TREK Channel Interactions with AKAP150 and Mtap2

Some K2P channel types have been found to have binding partners that influence channel function as well as potentially regulating trafficking of the channel to the plasma membrane [62]. An identified binding partner of TREK1 channels is the A kinase anchoring protein 150 (AKAP150) a scaffold protein [73], which does not have a direct trafficking role, but is important for tethering of proteins into complexes for signalling (Table **1**). Binding of AKAP150 to the regulatory domain in the C terminus of TREK1 channels, switches the channel from a low open probability, outwardly-rectifying conductance to a higher open probability leak conductance. More recently, Sandoz *et al.* [72] have found that, in addition to AKAP150, TREK1 (and TREK2) channels have a distinct binding site on their C termini for the microtubule associated protein, Mtap2 which, when bound, enhances both channel surface expression and current density (Table **1**). Mtap2 is primarily found postsynaptically, in dendritic spines and dendrites, so it may act to localise TREK1 surface expression in these regions through selective microtubule-based transport. Once localised at these regions, TREK1 is placed at the centre of a complex network of regulatory proteins through its interactions with the scaffolding proteins AKAP150 and Mtap2 [72].

#### 3.4.2 TWIK Channels, Arf6/EFA6 and SUMO

A known binding partner of TWIK1 channels is EFA6, an exchange factor for ADP-ribosylation factor 6 (ARF6, [18]). The ARF6 family of small GTPases are involved in clathrin-independent endocytosis of membrane proteins, including, for example, M2 muscarinic acetylcholine receptors [16]. TWIK1 is highly expressed in renal proximal tubules and was found, using immunofluorescent tagging, to localise in the pericentriolar recycling endosomes [15]. Concurrent binding of Arf6, EFA6 and TWIK1 leads to increased internalisation of the channel (Table **1**). Upon internalisation, the vesicles are transferred to early endosomes and are integrated into the classical clathrin coated recycling pathway. The mechanism by which other K2P channels are later internalised and recycled has, thus far, not been elucidated.

TWIK channels have also been linked to the small ubiquitin related modifier protein, SUMO, and it has been suggested that sumoylation is necessary for functional expression of the channel at the plasma membrane ([63], Table **1**). However more recent work has questioned the importance of this mechanism [22] and suggests, instead, that TWIK1 channels are rapidly retrieved from the membrane and internalised through a dynamin dependent mechanism [23].

#### 3.4.3 TASK1 Channels and NOX4

In addition to interacting with trafficking proteins, it has been shown that TASK1 channels interact with NADPH oxidase 4 (NOX4) to confer oxygen sensitivity to TASK1 and mediate the oxygen-sensitive K current response in carotid and neuro-epithelial bodies [21, 41]. Recently it has been established that there is a direct interaction between these two proteins to mediate this effect [60]. It is of interest that TASK1 is neuroprotective under ischemic conditions [51].

## 4. K2P CHANNEL TRAFFICKING AND DISEASE

K2P channels have been identified as important in an increasing number of physiological and pathophysiological conditions. For example TASK channels have importance in anesthesia, respiration and hormone secretion, whilst TREK channels are important in heat and mechanical pain sensation, neuroprotection and mood regulation [5]. Furthermore, there is a suggested role for TASK1 channels in regulating the function of the HIV-1 accessory protein Vpu-1 ([30], Table **1**).

K2P channel activity has been implicated in a number of neuronal disease states as indeed has the expression and activity levels of a number of their potential chaperone proteins. Cancers (both within and out with the CNS), neuroprotection and nociception are just three examples of clinical situations in which failure or amplification of K2P channel trafficking might contribute to the disease state.

For each of these three disease states, we describe evidence, below, to show that up or down regulation of K2P channel activity contributes to the disease state. Interestingly, in each case, changes in known K2P channel chaperone proteins produce effects consistent with a change in K2P channel trafficking. Crucially, however, at this stage and in each case, direct evidence is lacking that the particular chaperone proteins and K2P channel subunits involved do, in fact, interact in these situations and that there is a causal relationship between alterations in K2P channel trafficking and the disease state itself.

### 4.1 Cancer

K channels have been shown to be directly involved in the signalling pathway that regulates oncogenesis. The direct involvement of these channels in oncogenesis is demonstrated when pharmacological blockade of K channel current induces an inhibition of cell proliferation in various human cancers [e.g.  55, 59, 81, 91]. The K2P channel, TASK3 seems to be important in this effect because an amplification of its gene expression is found in breast, lung, colon, and metastatic prostate cancers [53]. A direct link between TASK3 channels and oncogenesis has been demonstrated by Pei *et al.* [61] who have found that a TASK3 dominant negative mutation could prevent the formation of tumour cells.

	Despite this link, contrary to normal cells that show a high surface and ER expression of TASK3 channels [96], the tumour cells have an especially high intracellular labelling compared to the membrane. This low TASK3 membrane expression could be due to a problem in TASK3 membrane trafficking which induces in this way an intracellular accumulation of TASK3. 

One possible explanation for this intracellular accumulation is that there is some impediment to the normal link between TASK3 channel and 14-3-3 protein. For example, a modification of the interaction site at the C-terminal region of TASK3 (pentapeptide motif, see above) might occur during translocation. This is unlikely, however, since Rusznak *et al.* [67] found no alteration in the TASK3-specific mRNA sequence of melanoma cells studied. Furthermore, various studies show that 14-3-3 protein is essential for the multiplication of cells [35, 83] and it is over expressed in brain tumors [11, 12]. The exchange factor EFA6 which binds to TWIK1 channels [15], leading to the internalisation of the channel, is also over expressed in various cancers [70]. Thus it may be an increased expression then a compensatory increased internalisation of TASK3 channels through EFA6 or a related protein that is observed in these studies. 

### 4.2 Neuroprotection

The TREK family of K2P channels play an important role in neuroprotection during cerebral ischemia. This action is due to lipidic compounds such as polyunsaturated fatty acid [39] or lysophospholipids [7] which are produced during ischemia that activates TREK and TRAAK channels. The induced neuron hyperpolarization protects against glutamate excitotoxicity, and against calcium entry into cells.

The chaperone protein, 14-3-3β is upregulated after ischemia and it too has an important neuroprotective effect [e.g.  40, 69]. Thus both K2P channel activity and the level of a chaperone protein that promotes K2P channel trafficking to the plasma membrane are increased during ischemia and have beneficial neuroprotective roles. 

### 4.3 Nociception

K2P channels, especially TREK1 [2], and TRESK [4], are expressed in sensory neurons, and are involved in polymodal pain perception. The retention factor p11 seems, also, to have a role in pain perception: a p11 deletion exclusively from nociceptive primary sensory neurons in mice induced an attenuation of acute pain behaviour, but no changes in inflammatory pain were observed [20, 25]. Thus both an increase in K2P channel activity and a decrease in the level of a chaperone protein that may inhibit K2P channel trafficking from the ER (but see above), act to reduce the perception of painful stimuli. 

## 5. CONCLUSIONS

The study of K2P channel trafficking is in its infancy, indeed, even for the few processes where there is detailed information, there is much controversy about underlying mechanisms. So, for example, whilst it is clear that the 14-3-3/COP1 system is important for trafficking TASK channels from the ER, there is not full agreement as to how this occurs. Similarly, whilst p11 seems certain to have a role in the trafficking of TASK1 channels, it is not clear whether it promotes or inhibits forward TASK1 channel trafficking. The perceived importance of K2P channel current-density in a number of diseases states including cancer, neuropathic pain and depression [4, 5, 43, 61] suggests that a better understanding of the mechanisms and regulations that underpin the trafficking of these channels to the plasma membrane and to localised regions therein, may considerably enhance the probability of future therapeutic advances in these areas. 

## Figures and Tables

**Table1:** Binding Partners of K2P Channels

Binding Partner	Channel	Putative Role	Reference
**14-3-3**	TASK1/ TASK3	Increases the surface expression of the channel	[ 57, 64, 95]
**14-3-3**	TRESK	Regulates calcineurin-mediated activation of the channel	[14]
**AKAP150**	TREK1	Increases current by binding to regulatory domain	[73]
**ARF6 / EFA6**	TWIK1	Enhance channel internalisation	[15]
**COP1**	TASK1/TASK3	Channel is retained in the ER	[56, 95]
**Mtap2**	TREK1	Enhances surface expression and current density	[72]
**NOX4**	TASK1	Confers O_2_ sensitivity on channel	[41, 60]
**p11**	TASK1	Modulates surface expression of the channel	[26, 57, 65, 95]
**SUMO**	TWIK1	‘Silences’ the channel	[63, but see 22, 23]
**Vpu**	TASK1	Abolishes channel current	[30]

## ABBREVIATIONS

4-AP: = 4-aminopyridine
AKAP150: = A kinase anchoring protein 150
Arf6: = ADP-ribosylation factor 6
ASIC: = Acid-sensitive ion channel
CASK: = Calcium/calmodulin-dependent serine kinase
COP1: = Coatomer protein complex 1
DLgA: = Drosophila disc large tumour suppressor
EFA6: = Exchange factor for Arf6
ER: = Endoplasmic reticulum
K2P: = Two pore domain potassium channel
KChAP: = K channel associated protein
KChIP: = Kv channel interacting protein
K_IR_: = Inward-rectifier K channel
K_V_: = Voltage-gated K channel
MAGUK: = Membrane-associated guanylate kinase
Mtap2: = Microtubule associated protein 2
NOX4: = NADPH oxidase 4
PDZ: = PSD95 / DlgA / zo-1 (acronym)
PSD95: = Postsynaptic density protein 95
SAP97: = Synapse associated protein 97
SNARE: = SNAP (soluble NSF attachment protein) receptor
SUMO: = Small ubiquitin related modifier protein
SUR: = Sulphonylurea receptor
SRP: = Signal recognition particle
TALK: = TWIK-related alkaline pH activated K channel
TASK: = TWIK-related acid sensitive K channel
TEA: = Tetraethyl ammonium
THIK: = Tandem pore domain halothane inhibited K channel
TRAAK: = TWIK-related arachidonic acid stimulated K channel
TREK: = TWIK-related K channel
TRESK: = TWIK-related spinal cord K channel
TRP: = Transient receptor potential
TWIK: = Tandem of P domains in a weak inward rectifier K channel
Vpu: = Viral protein U
Zo-1: = Zonula occludens-1 protein

## References

[1] Alexander SPH, Mathie A, Peters JA (2009). Guide to Receptors and Channels (GRAC). Br. J. Pharmacol.

[2] Alloui A, Zimmermann K, Mamet J, Duprat F, Noël J, Chemin J, Guy N, Blondeau N, Voilley N, Rubat-Coudert C, Borsotto M, Romey G, Heurteaux C, Reeh P, Eschalier A, Lazdunski M (2006). TREK-1, a K+ channel involved in polymodal pain perception. EMBO J.

[3] An WF, Bowlby MR, Betty M, Cao J, Ling HP, Mendoza G, Hinson JW, Mattsson KI, Strassle BW, Trimmer JS, Rhodes KJ (2000). Modulation of A-type potassium channels by a family of calcium sensors. Nature.

[4] Bautista DM, Sigal YM, Milstein AD, Garrison JL, Zorn JA, Tsuruda PR, Nicoll RA, Julius D (2008). Pungent agents from Szechuan peppers excite sensory neurons by inhibiting two-pore potassium channels. Nat. Neurosci.

[5] Bayliss DA, Barrett PQ (2008). Emerging roles for two-pore-domain potassium channels and their potential therapeutic impact. Trends Pharmacol. Sci.

[6] Bethune J, Wieland F, Moelleken J (2006). COPI-mediated transport. J. Memb. Biol.

[7] Blondeau N, Lauritzen I, Widmann C, Lazdunski M, Heurteaux C (2002). A potent protective role of lysophospholipids against global cerebral ischemia and glutamate excitotoxicity in neuronal cultures. J. Cereb. Blood Flow Metab.

[8] Brickley SG, Aller MI, Sandu C, Veale EL, Alder FG, Sambi H, Mathie A, Wisden W (2007). TASK-3 two-pore domain potassium channels enable sustained high-frequency firing in cerebellar granule neurons. J. Neurosci.

[9] Brock C, Boudier L, Maurel D, Blahos J, Pin J P (2005). Assembly-dependent surface targeting of the heterodimeric GABAB receptor is controlled by COPI but not 14-3-3. Mol. Biol. Cell.

[10] Campomanes CR, Carroll KI, Manganas LN, Hershberger ME, Gong B, Antonucci DE, Rhodes KJ, Trimmer JS (2002). J. Biol. Chem.

[11] Cao L, Cao WD, Zhang W, Lin H, Yang X, Zhen HN, Cheng J, Dong W, Huo J, Zhang X (2008). Identification of 14-3-3 protein isoforms in human astrocytoma by immunohistochemistry. Neurosci. Lett.

[12] Cao WD, Zhang X, Zhang JN, Yang ZJ, Zhen HN, Cheng G, Li B, Gao D (2006). Immunocytochemical detection of 14-3-3 in primary nervous system tumors. J. Neurooncol.

[13] Clarke CE, Veale EL, Wyse K, Vandenberg JI, Mathie A (2008). The M1P1 loop of TASK3 K2P channels apposes the selectivity filter and influences channel function. J. Biol. Chem.

[14] Czirjak G, Vuity D, Enyedi P (2008). Phosphorylation-dependent binding of 14-3-3 proteins controls TRESK regulation. J. Biol. Chem.

[15] Decressac S, Franco M, Bendahhou S, Warth R, Knauer S, Barhanin J, Lazdunski M, Lesage F (2004). ARF6-dependent interaction of the TWIK1 K+ channel with EFA6, a GDP/GTP exchange factor for ARF6. EMBO Rep.

[16] Delaney K A, Murph M M, Brown L M, Radhakrishna H (2002). Transfer of M2 muscarinic acetylcholine receptors to clathrin-derived early endosomes following clathrin-independent endocytosis. J. Biol. Chem.

[17] Deutsch C (2002). Potassium channel ontogeny. Ann. Rev. Physiol.

[18] Deutsch C (2003). The birth of a channel. Neuron.

[19] Donaldson J G (2003). Multiple roles for Arf6: Sorting, structuring, and signaling at the plasma membrane. J. Biol. Chem.

[20] Donier E, Rugiero F, Okuse K, Wood J N (2005). Annexin II light chain p11 promotes functional expression of acid-sensing ion channel ASIC1a. J. Biol. Chem.

[21] Duprat F, Lauritzen I, Patel A, Honore E (2007). The TASK background K2P channels: chemo- and nutrient sensors. Trends Neurosci.

[22] Feliciangeli S, Bendahhou S, Sandoz G, Gounon P, Reichold M, Warth R, Lazdunski M, Barhanin J, Lesage F (2007). Does sumoylation control K2P1/TWIK1 background K+ channels?. Cell.

[23] Feliciangeli S, Tardy MP, Sandoz G, Chatelain F, Warth R, Barhanin J, Bendahhou S, Lesage F (2010). Potassium channel silencing by constitutive endocytosis and intracellular sequestration. J. Biol. Chem.

[24] Fink M, Duprat F, Lesage F, Heurteaux C, Romey G, Barhanin J, Lazdunski M (1996). A new K+ channel beta subunit to specifically enhance Kv2.2 (CDRK) expression. J. Biol. Chem.

[25] Foulkes T, Nassar MA, Lane T, Matthews EA, Baker MD, Gerke V, Okuse K, Dickenson AH, Wood JN (2006). Deletion of annexin 2 light chain p11 in nociceptors causes deficits in somatosensory coding and pain behavior. J. Neurosci.

[26] Girard C, Tinel N, Terrenoire C, Romey G, Lazdunski M, Borsotto M (2002). p11, an annexin II subunit, an auxiliary protein associated with the background K+ channel, TASK-1. EMBO J.

[27] Goldstein SAN, Bayliss DA, Kim D, Lesage F, Plant LD, Rajan S (2005). International union of pharmacology. LV. Nomenclature and molecular relationships of two-P potassium channels. Pharmacol. Rev.

[28] Gulbis JM, Mann S, MacKinnon R (1999). Structure of a voltage-dependent K+ channel beta subunit. Cell.

[29] Honore E (2007). The Neuronal background K2P channels: focus on TREK1. Nat. Rev. Neurosci.

[30] Hsu K, Seharaseyon J, Dong P, Bour S, Marban E (2004). Mutual functional destruction of HIV-1 vpu and host TASK-1 channel. Mol. Cell.

[31] Jing J, Chikvashvili D, Singer-Lahat D, Thornhill WB, Reu-veny E, Lotan I (1999). Fast inactivation of a brain K+ channel composed of Kv1.1 and Kvbeta1.1 subunits modulated by G protein beta gamma subunits. EMBO J.

[32] Johnson AE, Van Waes MA (1999). The translocon: a dynamic gateway at the ER membrane. Annu. Rev. Cell. Dev. Biol.

[33] Kim D (2005). Physiology and pharmacology of two-pore domain potassium channels. Curr. Pharm. Des.

[34] Kim LA, Furst J, Butler MH, Xu S, Grigorieff N, Goldstein SA (2004). Ito channels are octomeric complexes with four subunits of each Kv4.2 and K+ channel-interacting protein 2. J. Biol. Chem.

[35] Komiya Y, Kurabe N, Katagiri K, Ogawa M, Sugiyama A, Kawasaki Y, Tashiro F (2008). A novel binding factor of 14-3-3beta functions as a transcriptional repressor and promotes anchor-age-independent growth, tumorigenicity, and metastasis. J. Biol. Chem.

[36] Kosolapov A, Deutsch C (2003). Folding of the voltage-gated K+ channel T1 recognition domain. J. Biol. Chem.

[37] Kuryshev YA, Gudz TI, Brown AM, Wible BA (2000). KChAP as a chaperone for specific K+ channels. Am. J. Physiol. Cell. Physiol.

[38] Lai HC, Jan LY (2006). The distribution and targeting of neuronal voltage-gated ion channels. Nat. Rev. Neurosci.

[39] Lauritzen I, Blondeau N, Heurteaux C, Widmann C, Romey G, Lazdunski M (2000). Polyunsaturated fatty acids are potent neuroprotectors. EMBO J.

[40] Lawrence EJ, Dentcheva E, Curtis KM, Roberts VL, Siman R, Neumar RW (2005). Neuroprotection with delayed initiation of prolonged hypothermia after *in vitro* transient global brain ischemia. Resuscitation.

[41] Lee YM, Kim BJ, Chun YS, So I, Choi H, Kim MS, Park JW (2006). NOX4 as an oxygen sensor to regulate TASK-1 activity. Cell. Signal.

[42] Li M, Jan YN, Jan LY (1992). Specification of subunit assembly by the hydrophilic amino-terminal domain of the Shaker potassium channel. Science.

[43] Lodge NJ, Li Y-W (2008). Ion Channels as potential targets for the treatment of depression. Curr. Opin. Drug Discov. Dev.

[44] Lotshaw DP (2007). Biophysical, pharmacological, and functional characteristics of cloned and native mammalian two-pore domain K+ channels. Cell Biochem. Biophys.

[45] Ma D, Jan L Y (2002). ER transport signals and trafficking of potassium channels and receptors. Curr. Opin. Neurobiol.

[46] Ma D, Zerangue N, Raab-Graham K, Fried SR, Jan YN, Jan LY (2002). Diverse trafficking patterns due to multiple traffic motifs in G protein-activated inwardly rectifying potassium channels from brain to heart. Neuron.

[47] Maffie J, Rudy B (2008). Weighing the evidence for a ternary protein complex mediating A-type K+ currents in neurons. J. Physiol.

[48] Mathie A (2007). Neuronal two-pore-domain potassium channels and their regulation by G protein-coupled receptors. J. Physiol.

[49] Mathie A, Veale EL (2007). Therapeutic potential of neuronal two-pore domain potassium-channel modulators. Curr. Opin. Invest. Drugs.

[50] Meuth SG, Budde T, Kanyshkova T, Broicher T, Munsch T, Pape HC (2003). Contribution of TWIK-related acid-sensitive K+ channel 1 (TASK1) and (TASK3) channels to the control of activity modes in thalamocortical neurons. J. Neurosci.

[51] Meuth SG, Kleinschnitz C, Broicher T, Austinat M, Braeuninger S, Bittner S, Fischer S, Bayliss DA, Budde T, Stoll G, Wiendl H (2009). The neuroprotective impact of the leak potassium channel TASK1 on stroke development in mice. Neurobiol. Dis.

[52] Mrowiec T, Schwappach B (2006). 14-3-3 proteins in membrane protein transport. Biol. Chem.

[53] Mu D, Chen L, Zhang X, See LH, Koch CM, Yen C, Tong JJ, Spiegel L, Nguyen KC, Servoss A, Peng Y, Pei L, Marks JR, Lowe S, Hoey T, Jan LY, McCombie WR, Wigler MH, Powers S (2003). Genomic amplification and oncogenic properties of the KCNK9 potassium channel gene. Cancer Cell.

[54] Nagaya N, Papazian DM (1997). Potassium channel alpha and beta subunits assemble in the endoplasmic reticulum. J. Biol. Chem.

[55] Nilius B, Wohlrab W (1992). Potassium channels and regulation of proliferation of human melanoma cells. J. Physiol.

[56] O’Kelly I, Butler MH, Zilberberg N, Goldstein SA (2002). Forward transport. 14-3-3 binding overcomes retention in endoplasmic reticulum by dibasic signals. Cell.

[57] O’Kelly I, Goldstein SAN (2008). Forward transport of K2P3.1: Mediation by 14-3-3 and COP1, modulation by p11. Traffic.

[58] Okuse K, Malik-Hall M, Baker M D, Poon W Y, Kong H, Chao M V, Wood JN (2002). Annexin II light chain regulates sensory neuron-specific sodium channel expression. Nature.

[59] Pancrazio JJ, Tabbara IA, Kim YI (1993). Voltage-activated K + conductance and cell proliferation in small-cell lung cancer. Anticancer Res.

[60] Park SJ, Chun YS, Park KS, Coi SO, Kim HL, Park JW (2009). Identification of subdomains in NADPH oxidase-4 critical for the oxygen-dependent regulation of TASK-1 K+ channels. Am. J. Physiol. Cell Physiol.

[61] Pei L, Wiser O, Slavin A, Mu D, Powers S, Jan LY, Hoey T (2003). Oncogenic potential of TASK3 (Kcnk9) depends on K+ channel function. Proc. Nat. Acad. Sci. USA.

[62] Plant L D, Rajan S, Goldstein S A (2005). K2P channels and their protein partners. Curr. Opin. Neurobiol.

[63] Rajan S, Plant L D, Rabin M L, Butler M H, Goldstein S A (2005). Sumoylation silences the plasma membrane leak K+ channel K2P1. Cell.

[64] Rajan S, Preisig-Muller R, Wischmeyer E, Nehring R, Hanley PJ, Renigunta V, Musset B, Schlichthörl G, Derst C, Karschin A, Daut J (2002). Interaction with 14-3-3 proteins promotes functional expression of the potassium channels TASK-1 and TASK-3. J. Physiol.

[65] Renigunta V, Yuan H, Zuzarte M, Rinne S, Koch A, Wisch-meyer E, Schlichthorl G, Gao Y, Karschin A, Jacob R, Schwappach B, Daut J, Preisig-Muller R (2006). The retention factor p11 confers an endoplasmic reticulum-localization signal to the potassium channel TASK-1. Traffic.

[66] Rescher U, Gerke V (2008). S100A10/p11: family, friends and functions. Pflugers Arch.

[67] Rusznák Z, Bakondi G, Kosztka L, Pocsai K, Dienes B, Fodor J, Telek A, Gönczi M, Szucs G, Csernoch L (2008). Mitochondrial expression of the two-pore domain TASK-3 channels in malignantly transformed and non-malignant human cells. Virchows Arch.

[68] Sabbadini M, Yost CS (2009). Molecular biology of background K channels: insights from K(2P) knockout mice. J. Mol. Biol.

[69] Saito A, Narasimhan P, Hayashi T, Okuno S, Ferrand-Drake M, Chan PH (2004). Neuroprotective role of a proline-rich Akt substrate in apoptotic neuronal cell death after stroke: relationships with nerve growth factor. J. Neurosci.

[70] Sakagami H (2008). The EFA6 family: guanine nucleotide exchange factors for ADP ribosylation factor 6 at neuronal synapses. Tohoku J. Exp. Med.

[71] Salinas M, Reyes R, Lesage F, Fosset M, Heurteaux C, Romey G, Lazdunski M (1999). Cloning of a new mouse two-P domain channel subunit and a human homologue with a unique pore structure. J. Biol. Chem.

[72] Sandoz G, Tardy MP, Thummler S, Feliciangeli S, Lazdunski M, Lesage F (2008). Mtap2 is a constituent of the protein network that regulates Twik-related K+ channel expression and trafficking. J. Neurosci.

[73] Sandoz G, Thummler S, Duprat F, Feliciangeli S, Vinh J, Escoubas P, Guy N, Lazdunski M, Lesage F (2006). AKAP150, a switch to convert mechano-, pH- and arachidonic acid-sensitive TREK K+ channels into open leak channels. EMBO J.

[74] Scannevin RH, Wang K, Jow F, Megules J, Kopsco DC, Edris W, Carroll KC, Lu, Xu W, Xu Z, Katz AH, Olland S, Lin L, Taylor M, Stahl M, Malakian K, Somers W, Mo-syak L, Bowlby MR, Chanda P, Rhodes KJ (2004). Two N-terminal domains of Kv4 K(+) channels regulate binding to and modulation by KChIP1. Neuron.

[75] Schwappach B (2008). An overview of trafficking and assembly of neurotransmitter receptors and ion channels. Mol. Membr. Biol.

[76] Schwappach B, Zerangue N, Jan YN, Jan LY (2000). Molecular basis for KATP assembly: transmembrane interactions mediate association of a K+ channel with an ABC transporter. Neuron.

[77] Shi G, Nakahira K, Hammond S, Rhodes KJ, Schechter  
LE, Trimmer JS (1996). B-subunits promote K+ channel surface expression through effects early in biosynthesis. Neuron.

[78] Shibata R, Misonou H, Campomanes CR, Anderson AE, Schrader LA, Doliveira LC, Carroll KI, Sweatt JD, Rhodes KJ, Trimmer JS (2003). A fundamental role for KChIPs in
determining the molecular properties and trafficking of Kv4.2 potassium
channels. J. Biol. Chem.

[79] Shikano S, Coblitz B, Sun H, Li M (2005). Genetic isolation of transport signals directing cell surface expression. Nat. Cell Biol.

[80] Shikano S, Coblitz B, Wu M, Li M (2006). 14-3-3 proteins: Regulation of endoplasmic reticulum localization and surface expression of membrane proteins. Trends Cell Biol.

[81] Skryma RN, Prevarskaya NB, Dufy-Barbe L, Odessa MF, Audin J, Dufy B (1997). Potassium conductance in the androgen-sensitive prostate cancer cell line, LNCaP: involvement in cell proliferation. Prostate.

[82] Steele DF, Eldstrom J, Fedida D (2007). Mechanisms of cardiac potassium channel trafficking. J. Physiol.

[83] Sugiyama A, Miyagi Y, Komiya Y, Kurabe N, Kitanaka C, Kato N, Nagashima Y, Kuchino Y, Tashiro F (2003). Forced expression of antisense 14-3-3beta RNA suppresses tumor cell growth *in vitro* and *in vivo*. Carcinogenesis.

[84] Svenningsson P, Chergui K, Rachleff I, Flajolet M, Zhang X, El Yacoubi M, Vaugeois JM, Nomikos GG, Greengard P (2006). Alteration in 5-HT1B receptor function by p11 in depression-like states. Science.

[85] Svenningsson P, Greengard P (2007). p11S100A10—an inducible adaptor protein that modulates neuronal functions. Curr. Opin. Pharmacol.

[86] Tu L, Deutsch C (1999). Evidence for dimerization of dimers in K+ channel assembly. Biophys. J.

[87] Tu L, Wang J, Helm A, Skach WR, Deutsch C (2000). Transmembrane Biogenesis of Kv1.3. Biochemistry.

[88] Veale EL, Buswell R, Clarke CE, Mathie A (2007). Identification of a region in the TASK3 two pore domain potassium channel that is critical for its blockade by methanandamide. Br. J. Pharmacol.

[89] Vivithanaporn P, Yan S, Swanson GT (2006). Intracellular
trafficking of KA2 kainate receptors mediated by interactions with
coatomer protein complex I (COPI) and 14-3-3 chaperone systems. J. Biol. Chem.

[90] Wible BA, Yang Q, Kuryshev YA, Accili EA, Brown AM (1998). Cloning and expression of a novel K+ channel regulatory protein, KChAP. J. Biol. Chem.

[91] Woodfork KA, Wonderlin WF, Peterson VA, Strobl JS (1995). Inhibition of ATP-sensitive potassium channels causes reversible cell-cycle arrest of human breast cancer cells in tissue culture. J. Cell Physiol.

[92] Yuan H, Michelsen K, Schwappach B (2003). 14-3-3 dimers probe the assembly status of multimeric membrane proteins. Curr. Biol.

[93] Zerangue N, Schwappach B, Jan YN, Jan LY (1999). A new ER trafficking signal regulates the subunit stoichiometry of plasma membrane K_ATP_ channels. Neuron.

[94] Zhou W, Qian Y, Kunjilwar K, Pfaffinger PJ, Choe S (2004). Structural insights into the functional interaction of KChIP1 with Shal-type K(+) channels. Neuron.

[95] Zuzarte M, Heusser K, Renigunta V, Schlichthorl G, Rinne S, Wischmeyer E, Daut J, Scwappach B, Preisig-Muller R (2009). Intracellular traffic of the K+ channels TASK-1 and TASK-3: role of N- and C-terminal sorting signals and interaction with 14-3-3 proteins. J. Physiol.

[96] Zuzarte M, Rinne S, Schlichthorl G, Schubert A, Daut J, Preisig-Muller R (2007). A Di-acidic sequence motif enhances the surface expression of the potassium channel TASK-3. Traffic.

